# Self-Directed Exergaming for Stroke Upper Limb Impairment Increases Exercise Dose Compared to Standard Care

**DOI:** 10.1177/15459683211041313

**Published:** 2021-08-27

**Authors:** Michelle Broderick, Leeza Almedom, Etienne Burdet, Jane Burridge, Paul Bentley

**Affiliations:** 1 Department of Brain Sciences, 4615 Imperial College London, Charing Cross Hospital Campus, London, UK; 2 Department. of Bioengineering, Human Robotics Group, Imperial College, South Kensington Campus, London, UK; 3 Department of Restorative Neuroscience, University of Southampton, Southampton, UK

**Keywords:** stroke, rehabilitation, physiotherapy, upper limb, exercise gaming, rehabilitation technology

## Abstract

*Background*. One of the strongest modifiable determinants of rehabilitation outcome is exercise dose. Technologies enabling self-directed exercise offer a pragmatic means to increase dose, but the extent to which they achieve this in unselected cohorts, under real-world constraints, is poorly understood. *Objective*. Here we quantify the exercise dose achieved by inpatient stroke survivors using an adapted upper limb (UL) exercise gaming (exergaming) device and compare this with conventional (supervised) therapy. *Methods*. Over 4 months, patients presenting with acute stroke and associated UL impairment were screened at a single stroke centre. Participants were trained in a single session and provided with the device for unsupervised use during their inpatient admission. *Results*. From 75 patients referred for inpatient UL therapy, we recruited 30 (40%), of whom 26 (35%) were able to use the device meaningfully with their affected UL. Over a median enrolment time of 8 days (IQR: 5–14), self-directed UL exercise duration using the device was 26 minutes per day (median; IQR: 16–31), in addition to 25 minutes daily conventional UL therapy (IQR: 12–34; same cohort plus standard care audit; joint n = 50); thereby doubling total exercise duration (51 minutes; IQR: 32–64) relative to standard care (Z = 4.0, P <.001). The device enabled 104 UL repetitions per day (IQR: 38–393), whereas conventional therapy achieved 15 UL repetitions per day (IQR: 11–23; Z = 4.3, P <.001). *Conclusion*. Self-directed adapted exergaming enabled participants in our stroke inpatient cohort to increase exercise duration 2-fold, and repetitions 8-fold, compared to standard care, without requiring additional professional supervision.

## Introduction

Upper limb (UL) impairment is the most common physical consequence of stroke,^1^ with ∼60% of stroke survivors experiencing persistent UL functional impairment.^2^ Repetitive task-directed exercise improves long-term UL recovery,^3,4^ making this a key component of occupational therapy (OT) and physiotherapy (PT) post-stroke management.

Higher exercise doses are associated with superior UL outcomes in animal stroke models^5,6^ and clinical trials,^7^ with a dose threshold emerging at approximately 2 hours active training^8^ and several hundred UL repetitions daily.^9^ In practice, the amounts of organized physical therapy provided to patients are relatively low.^10^ A UK audit found that OT and PT provision nationally falls below recommended guidelines for post-stroke rehabilitation (45 minutes daily over 5 days), with centres delivering on average 40 minutes/day OT across 65% inpatient days and 35 minutes/day PT over 73% inpatient days (equivalent to 28 and 25 minutes daily, respectively).^11^ A review of observational studies of inpatient stroke therapy indicated that the average UL treatment component for OT and PT sessions combined, lasts ∼10 minutes and comprises ∼30 repetitions.^12^ The reasons why practice falls far behind theoretically optimal levels of exercise dosage are multifaceted, including barriers such as costs and staffing^13,14^ as well as reduced capacity of stroke survivors to initiate and engage in self-directed exercise^15^ (defined as when ≥50% of training occurs outside of direct professional supervision^16^).

In recent years, a growing number of rehabilitation technologies have emerged that boast the potential to provide cost-effective, intensive UL exercise.^17^ However, while such devices provide the *means* to supplement exercise dose, their clinical adoption, thus far, has been underwhelming.^18^ Bridging this ‘translational gap’ is a much needed focus of rehabilitation research; requiring design optimization (cost, complexity, accessibility etc.)^19^ and tests of patient engagement and efficacy.^20,21^

One such knowledge gap concerns the extent to which UL rehabilitation technologies can be adopted in real-world, heterogeneous populations, including those with severe weakness, cognitive impairment etc. Studies of rehabilitation technologies to date have typically selected high-functioning cohorts, which limits the applicability of their findings.^22^ A related issue is that clinical trials of UL rehabilitation technologies typically control for exercise dosage between treatment groups, in order to test whether interventions are as effective as dose-matched, conventional therapy.^23-28^ This entails researchers imposing scheduled therapy sessions, while closely supervising and supporting participants. However, this confounds one of the main attractions of rehabilitation technologies: that is, the potential to increase exercise dose without additional cost or manpower. Therefore, to maximize translational potential, trials of rehabilitation technologies should be subject to the constraints of typical healthcare settings, including the need to match professional contact time (rather than exercise dose) between intervention and standard care cohorts. In such studies, exercise dose achieved becomes an important outcome measure of interest.

Here we trial a self-directed exergaming technology, adapted (or customized) for stroke survivors,^29^ distinguishing it from commercial technologies (Laver et al 2017). The intervention is self-directed in the sense that patients can use it without formal support or supervision, although may be assisted by informal carers eg family. Exergaming refers to ‘*digital games that require bodily movements to play, stimulating an active gaming experience to function as a form of physical activity’*.^30^ Exergaming interventions may also be categorized as either interactive gaming or non-immersive virtual reality^31^ The purpose of the current study was to estimate: i) the amount of supplementary exercise that can be achieved using an adapted exergaming device designed for self-directed UL exercise and ii) to compare this with exercise dose achieved by conventional, supervised therapy, within a standard hospital setting. The study follows a broad cohort of stroke survivors with UL impairment in the subacute recovery phase, when neurobiological recovery potential is heightened and rehabilitative therapy is typically concentrated. By testing a heterogeneous sample, we also address the question of iii) how technology-enabled, self-directed exercise supplementation depends upon patient characteristics including physical and cognitive impairment.

A further important divergence from many previous UL rehabilitation technology trials is that our study is relatively low-resource and ‘hands-off’. In this way, we maximize its applicability to everyday practice, where finding extra budget and personnel to adopt and implement novel interventions is a common barrier. Accordingly, the device we employ is low cost and designed for self-directed exercise across a broad range of user abilities.^29^

## Methods

### Ethics

The study was approved by the UK National Research Ethics Service (Ref: 78 462). All participants gave informed written consent prior to recruitment.

### Study Design

We conducted a prospective feasibility study of a self-directed adapted exercise gaming device for UL impairment. The device was provided as an adjunct to conventional therapy in a cohort of subacute stroke in-patients with new UL impairment. Outcome measures were as follows: feasibility and fidelity of the research protocol; accessibility and acceptability of the device; supplementary UL exercise dose (duration and repetitions) achieved and the influence of participant characteristics on both participation and performance.

UL exercise dose achieved with the device was compared with that recorded during conventional therapy in the same set of patients and study period. This enabled estimation of both the supplementary and total exercise dose achieved in subjects provided with the device, who also received standard care. Conventional therapy was not intended to be altered by, nor incorporate device use. To confirm this, we also conducted a prospective audit of therapy content and duration in a separate cohort of patients with similar characteristics, receiving standard care only.

### Study Setting

The study took place at a large London stroke centre (∼1200 admissions p.a.) between September and December 2019. Conventional therapy provision at this centre was 36.6 minutes daily (IQR: 23.0–61.7) for OT and 37.2 minutes for PT. This was for all rehabilitation domains, across all 627 admitted stroke patients during the trial and a subsequent audit period (September 2019–February 2020. This indicates that conventional care dose in our population complied with national therapy guidelines and exceeded the national average.^13^

### Patient Population

All patients presenting with UL impairment suitable for inpatient therapy were screened. In the following two months (January–February 2020), an audit of patient medical records was conducted in a cohort of stroke in-patients with similar characteristics. Inclusion criteria consisted of: i) adults admitted with acute stroke within previous 4 weeks; ii) objective UL weakness due to presenting stroke; iii) capacity to consent. Exclusion criteria consisted of: i) medical instability; ii) unremitting UL pain (any aetiology, self-reported or clinically indicated); iii) uncompensated visual impairment; iv) significant language or communication barrier; v) photosensitive epilepsy and vi) participation in a concurrent research trial.

### Intervention

All participants were provided with the adapted exergaming device from enrolment for the remainder of their inpatient admission within the study centre. Patients were taught to use the device by a research therapist in a single ∼40-minute session. Training employed a standardized script, including dose-response education; management of compensatory or accessory movements and safety/adverse events reporting procedures. Participants were encouraged to use the device daily with their affected UL. Relatives or friends were engaged, if available and required, for example, for device positioning/set up. Personalized recommendations were also issued, for example, use of UL positioning support and selection of suitable training games. After the first training session, participants (with or without non-professional assistance) were left to use the device freely without coercion, prompting or professional supervision. A research team member visited weekly to screen for and address any technology support needs; between visits, participants and delegated clinicians were encouraged to contact the research team via phone or email if required.

**Figure 1.**
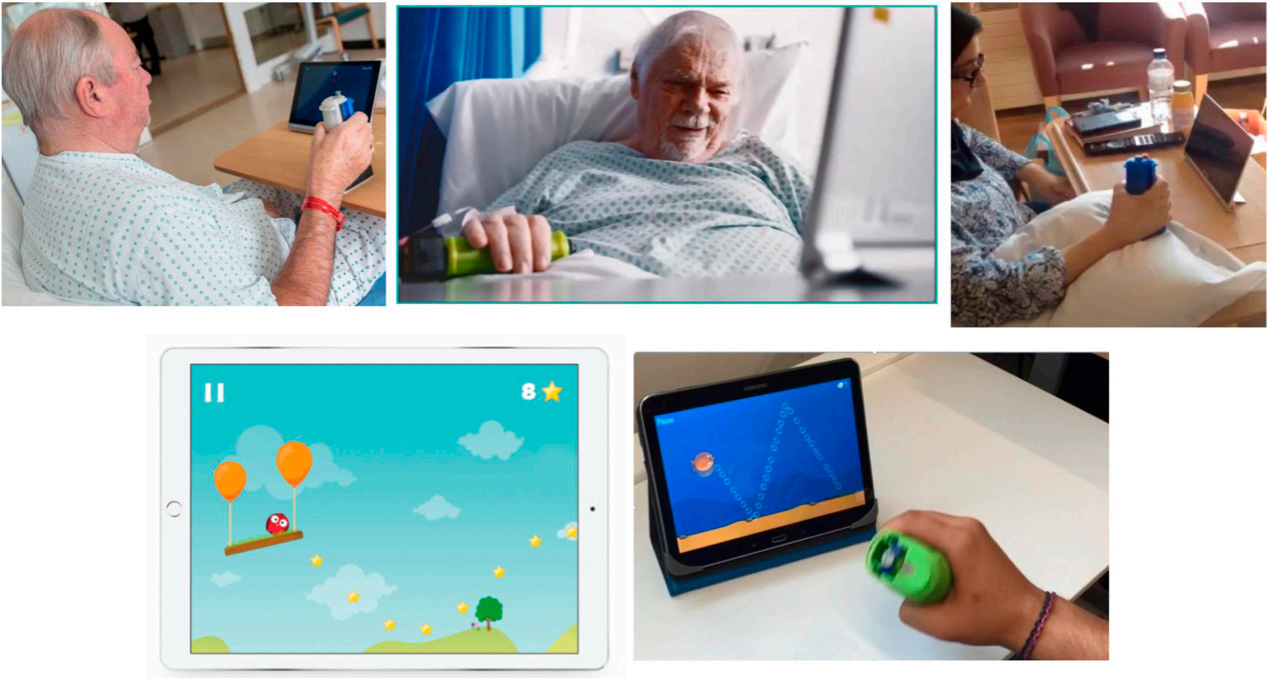
Examples of stroke in-patients using the adapted exergaming system. Exergames trained (i) finger flexion and release (e.g. here shown controlling the height of a balloon, so as to steer the bird on the beam into the path of the stars) and (ii) wrist (here, showing pronation – supination). Full consent was sought from participants for use of these images for publication and research dissemination purposes.

The adapted exergaming device consists of a handheld flexible sensor system that detects both grip force and 6 degrees-of-freedom acceleration, enabling training of precision grip control, finger extension and wrist movements.^29^ The sensor system communicates wirelessly with a tablet on which there are 8 training games providing feedback that participants select at will (Figure 1). At the start of each session, participants were prompted by the software to exert a maximal force grip; this calibrated the exergaming activities, enabling engagement across a wide range of motor abilities.

### Measures

*1. Patient characteristics:* The following data were recorded at study entry: age; sex; Edinburgh Handedness Scale (EHS); stroke type (ischemic/haemorrhagic); National Institute of Health Stroke Scale (NIHSS); NIHSS arm-motor subcomponent; Fugl Meyer-Upper Extremity Assessment (FM-UE); Montreal Cognitive Assessment (MoCA); modified Rankin Scale (mRS); Hospital Anxiety and Depression Scale (HADS) and Barthel Index (BI).

2. *Feasibility and fidelity:* We recorded: (i) fatigue and pain (using the Fatigue Severity Scale and Faces Pain Rating Scale at enrolment and end point. During the intervention period, fatigue and pain were monitored and recorded via screening questions, clinician observations and/or participant self-report.); (ii) adverse events; (iii) technical failure and support required and (iv) recruitment and retention rates. At study end point, a feedback survey was administered to evaluate the acceptability of the research protocol, research materials and research process. This incorporated a 5-point Likert scale (dissatisfied, room for improvement, neutral, satisfied, and extremely satisfied), and a single binary question: willingness to be randomized to a future trial (yes/no).

*3. Accessibility and acceptability:* The accessibility of the device for each participant was rated by the research therapist on enrolment, using a 4-point user competency scale: independent; support for set up only; supervision and support required or unable to use meaningfully. Competency judgements were made based on the following device functionalities: set up; turning on; accessing the exercise game platform; selecting and executing exercise software and device charging.

Acceptability of the device was evaluated using an 11‐item survey based on the Technology Acceptance Model (TAM)^32^ that is predictive of novel healthcare technology adoption^33,34^. Items measured included perceived usefulness, intentions to use and perceived ease of use. Participants indicated their level of agreement with each item on a 3-point Likert scale (disagree, neutral, agree). Technology acceptance was indicated by a >75% positive response to affirmative survey items. Participants’ comments or supporting statements in the context of their feedback were also recorded.

*4. Supplementary dose of UL exercise:* Conventional UL therapy doses were extracted from: i) patient electronic clinical records that describe detailed content of each therapy session and ii) the UK Sentinel Stroke National Audit Programme (SSNAP) database, which itemizes therapy duration and frequency for individual patients. We also recorded whether, as part of conventional therapy, participants had an UL goal documented; whether they were provided with an UL self-exercise training programme (Graded Repetitive Arm Supplementary Programme (GRASP))^35^ and surveyed patients (from the audited group not receiving the device) as to the frequency with which GRASP was used and the conditions in which it was used (i.e. self-directed or supervised).

Daily durations of exergaming device use were logged both by patients/carers (i.e. start–end times of session) and by the device (i.e. cumulative time on exercise trials). These two logs were strongly correlated (intraclass correlation for absolute agreement: r = .87;; P *<*.001) indicating reliability of both measures. However, overall session times included preparatory steps (e.g. device positioning; set up and game selection) and rests that were not recorded by the device (which only logged active game play) and so the former were 14.5% greater (median; IQR: −.06 to 20.9%). Since preparatory and rest periods were included within conventional therapy session times, which correspond to ‘time scheduled for therapy’, as conventionally reported in rehabilitation studies,^8^ we use session times for direct comparison between the two therapy types.

In a subgroup of 11 participants we measured repetition counts during both conventional therapy and self-directed exergaming sessions. The age, NIHSS, FM-UE scores and enrolment duration were not significantly different in this subsample to the remainder of the group. During conventional therapy sessions, UL exercise repetition numbers were documented by therapists in clinical records. In order to assess their accuracy, a researcher directly observed conventional UL therapy sessions and counted UL repetitions in audited standard care patients (n = 15) and compared this to counts itemized in clinical records. Since this showed that actual repetition counts were 15% greater than those documented, we corrected the latter accordingly. Repetition numbers per session of continuous exercise; and per day; and exergame type, were electronically recorded by the device during self-directed UL exercise sessions.

### Statistical Analysis

Statistical analyses comprised between-group comparisons of: (1) conventional therapy time and (2) total therapy time, comparing subjects provided with the exergaming device (n = 30) vs those in the standard care audit (n = 20). For comparison of repetition counts, we performed both a between-group comparison (using standard care audit data) and a within-subject comparison for the subgroup of patients provided with the exergue device in whom we also recorded conventional therapy repetitions (n = 11).

Since study enrolment duration in days varied across patients, we tested the above comparisons both per patient and per day (collapsed over subjects). As one of the distributions of interest (therapy time per day) was non-normal (Kolmogorov statistic: .27; P <.001), we report medians, interquartile ranges and conduct non-parametric statistical analyses (Wilcoxon rank-sum for between-subject; Wilcoxon signed-rank for within-subject analyses and Spearman’s rank correlation), using MATLAB v.2019b.

## Data Availability

Datasets of the current study are available from the corresponding author upon reasonable request.

## Results

### Recruitment

**Figure 2.**
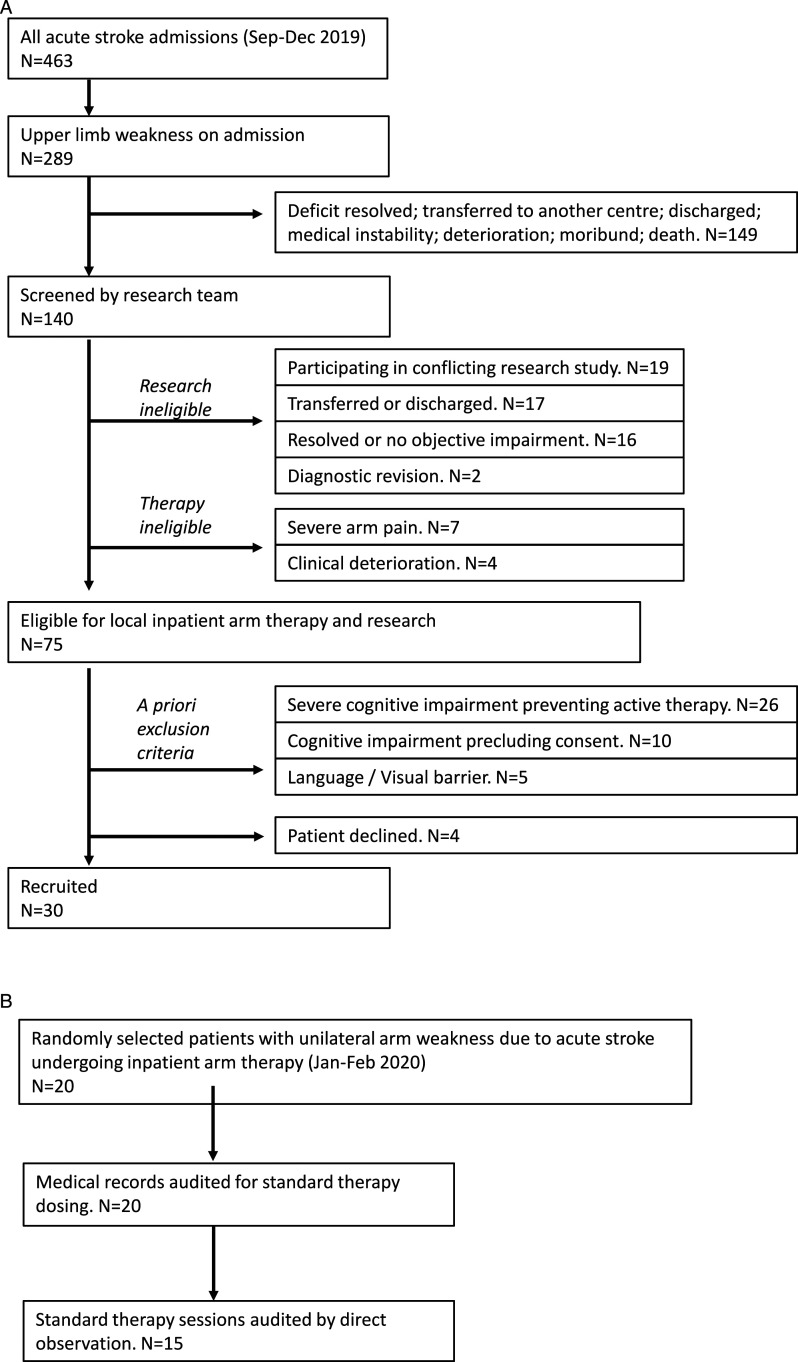
Flow charts showing numbers of patients screened vs recruited into intervention trial (A) and audited as part of standard care (B).

140 patients with UL impairment were screened, of whom 65 were unsuitable for research or therapy (Figure 2). Of the remaining 75, we recruited 30 participants (i.e. 40%), the main reason for exclusion was severe cognitive impairment (inability to provide informed consent). Subjects were enrolled for a median of 8 days (IQR: 5–14). The total number of enrolment days across all subjects was 381.

### Participant Characteristics

Participant characteristics of those recruited for intervention, and those audited for standard care, are detailed in Table 1. There were no significant differences in any measures between the two groups.

**Table 1.** Participant Characteristics.

	Device (+Standard Care)		Standard Care (Audit)		Effect Size (Cohen’s d equivalent¥)
	Median or n	IQR	Median or n	IQR	
**N**	30		20		
**Age/years**	72.5	61–79	63	54–76	.44
**Sex (female)/n**	14 (47%)		14 (70%)		.42
**Hemorrhagic stroke**	5 (17%)		6 (30%)		.24
**Premorbid functional dependency (mRS)/0–6**	1	0–2	1	1–3	.32
Delay between stroke onset and enrolment*/days	8.5	5–14	8	2–10	.61
**Duration of enrolment/days**	8	5–14	12	7.5–17	.45
**Total admission duration/days**	18.5	14–38	26	13–37.5	.05
**Global neurological disability (NIHSS)/0–42**	6	2–10	7	4–14	.41
**Arm weakness (R or L) (NIHSS subcomponent)/0–4**	2	1–3	1.5	1–2.5	.13
**Arm function (Fugl-Meyer Score)/0–66**	40	30–45	36.5	18–54	.17
**Cognitive score (MoCA)/0–30**	22	18–24	23	11–29	.21
**Rehabilitation goal set addressing arm recovery**	11 (37%)		12 (60%)		.42
**Provided with GRASP self-exercise programme**	17 (57%)		14 (70%)		.24
**Discharge functional dependency (mRS)/0–6**	3	3–4	4	3–4	.33

Abbreviations: mRS: modified Rankin Score; NIHSS: National Institute for Health Stroke Score; MoCA: Montreal Cognitive Assessment; GRASP: Graded Repetitive Arm Supplementary Programme; n.s.: not significant. Units or score range given. NIHSS and mRS scores are higher for worse disability. Fugl-Meyer and MoCA scores are lower for worse disability. * data collection for audit was started at delays from onset matching those of patients in device trial whose inpatient therapy duration was most closely matching. ¥ Effect sizes were calculated as AUROCC that is appropriate for non-parametric tests and then converted to Cohen d effect size equivalent (Harald Hentschke (2021). Hhentschke/measures-of-effect-size-toolbox (https://github.com/hhentschke/measures-of-effect-size-toolbox), GitHub. Retrieved March 16, 2021.; Salgado, Jesus (2018). Transforming the Area under the Normal Curve (AUC) into Cohen’s d, Pearson’s r pb, odds ratio and natural log odds ratio: Two Conversion Tables. The European Journal of Psychology Applied to Legal Context. 10.35-47.l10.5093/ejpalc2018a5. Statistical comparisons of the two groups were non-significant (P<.05) for every measure in the Table, using rank-sum for metric and ordinal data and chi-squared for proportions.

### Feasibility and Fidelity

Adverse events were monitored, recorded and assessed by the study chief investigator. There were no reported adverse or serious adverse events as per Good Clinical Practice monitoring and reporting standards,^36^including no self-reported or screened incidence of UL pain during the intervention period and no significant increase in pain $\left(t_{28}=1.99\right)$ or fatigue scores $\left(t_{28}=0.47\right)$ from baseline to end. Three episodes of device failure occurred requiring (remote) technical support. There were 20 reported episodes of minor technical errors, primarily relating to Bluetooth connectivity between device and tablet, which led to periods of disuse. These were resolved by the research therapist.

All participants rated the research protocol, information provided, consenting process, assessments completed and researcher visits as ‘satisfactory’ or ‘extremely satisfactory’. 17% (95% CI: 3–30%) rated the intervention training protocol as leaving ‘room for improvement’. 83% (95% CI: 70–97%) of participants cited willingness to be randomized in a future trial.

### Accessibility and Acceptability

4 participants (13%; 95% CI: 1–26%) were unable to use the device with their affected UL due to dense weakness (Medical Research Council Muscle Power Scale 0/5), that persisted throughout enrolment (verified by weekly review or consultation with the treating clinical team). Device user competence varied as follows: 7 (23%; 95% CI: 8–39%) participants were fully independent; 7 (23%; 95% CI: 8–39%) required assistance with set up only; 11 (37%: 95% CI: 19–54%) required supervision from a non-professional (e.g. friend or relative) due to physical/cognitive difficulties.

Across all participants (including those unable to use the device) the technology acceptance rating was 77% (95% CI: 62–92%), with 73% (95% CI: 58–89%) reporting that they would have liked to continue using the device (intent to use). 57% (95% CI: 39–74%) found the device easy to use and understand (perceived ease of use). 63% (95% CI: 46–81%) felt that the device promoted UL recovery (perceived usefulness). NIHSS was found to significantly correlate with technology acceptance (95% CI: −.56 [-.79,-.22] Adjusted P = .01). A non-significant correlation was found between FM-UE score and technology acceptance (r: −.39 [95% CI: .00, .66]; P = .08).

### Supplementary Exercise

An UL therapy goal was documented for 11/30 (37%; 95% CI: 19–54%) participants. The self-exercise programme, GRASP, was provided by therapists to 31/50 (62%; 95% CI: 49–76%) UL-impaired patients (pooling recruited and audited samples). A survey of 14 participants from the audited group indicated that GRASP was used exclusively in supervised therapy sessions in 79% (95% CI: 57–100%) and 1–2x/week independently in 21% (95% CI: 0–43%).

**Figure 3.**
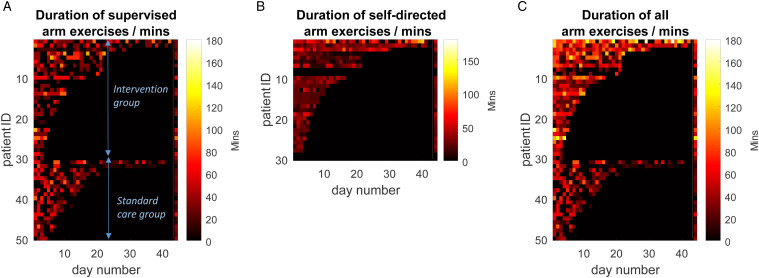
Heatmaps of daily arm exercise duration, with colour intensity indicating time (colorbar), broken down by exercise supervised by therapist (A); self-directed exercise using gaming device (B) and total time (C: i.e. = A+B). Patients are grouped, with the first 30 being those who received self-directed gaming device (i.e. intervention) and the second 20 being a standard care sample. Within these groups, patients are ranked by the number of inpatient days they received supervised therapy. Final column of each heatmap indicates subjects’ median daily exercise time.

Focussing on UL rehabilitation (combining OT and PT), there was no significant difference in daily conventional (i.e. supervised) therapy time between participants receiving the exergaming device (median: 31.2 minutes, IQR: 0–37.3; n = 30) vs those in the standard care audit (median: 23.3 minutes, IQR: 15.0–30.0; n = 20) (Z = .46, P *=* .647). Pooling device and standard care groups, the median daily conventional UL therapy duration was 25.0 minutes (IQR: 11.9–34.2) per participant or 15.0 minutes (IQR: 0–37.3) per day (Figure 3A). The additional median daily UL exercise duration achieved by participants using the exergaming device (including those unable to use the device) was 26.3 minutes per patient (IQR: 16.0–31.0) (Figure 3B) or 24.5 minutes (IQR: 7.0–37.0) per day. The total daily exercise duration (i.e. supervised + self-directed) for participants included in the study was 51.0 minutes (IQR: 31.0–62.5) or 45.0 minutes per day (IQR: 19.3–69.7), both of which were significantly greater than standard care (Z >3.8, P <.001) (Figure 3C).

**Figure 4.**
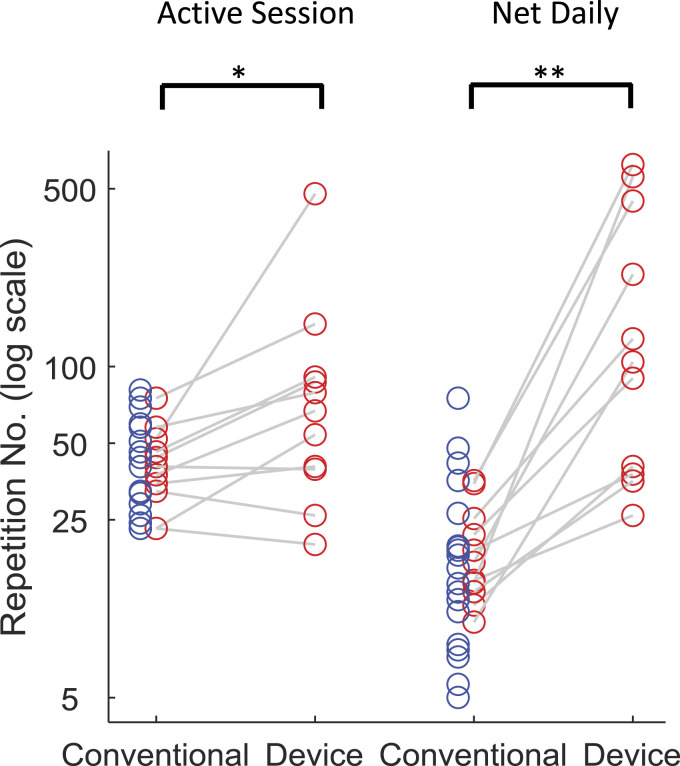
Arm exercise repetition counts comparing conventional therapy with device-assisted self-exercise: (A) median repetitions per session; (B) median repetitions per day per patient across all days measured within active therapy period (i.e. net daily = total repetitions/days). Blue circles refer to standard care (audited) patients; red circles refer to patients provided with device who underwent both conventional therapy and device-assisted self-directed exercise. Conventional counts are corrected for under-reporting. * P <.05; **P <.001

There was no significant difference in exercise repetition count during conventional therapy comparing participants provided with the device (n = 30) vs those in the standard care audit (n = 20): 40 (IQR: 33–52) vs 36 (IQR: 27–54) per session or 15 (IQR: 12–23) vs 15 (IQR: 8–23) per day (Z = .21, .39; P = .836, .695, respectively). Participants using the device achieved ∼60% more repetitions during self-directed (exergaming) training sessions (median: 67; IQR: 40–90; n = 11) than during their conventional therapy sessions (within-subject comparison; P = .019) or during conventional therapy sessions of the standard care group (between-group comparison: Z = 1.8, P = .066) (Figure 4). Measured as repetition counts per day, the difference in repetitions counts between participants using the device, during device sessions, vs all participants during conventional therapy sessions, was ∼7x greater (median: 104; IQR: 38–393 vs 15; IQR: 11–23; within-subject and between-group comparisons both: P<.001). The median number of exercise types selected by participants using the device was 3 (IQR: 2–6).

### Dependency on Baseline Characteristics

There was a non-significant for the correlation of UL impairment severity (FM-UE) and exercise gaming duration (FM-UE: Spearman r = .32, P = .082). Visual inspection of data suggested the largest effect was between subjects in the lowest tertile of UL ability (FM-UE) score 0–25) who used the device daily for 14.3 minutes (IQR: 0–30), vs more abled subjects (FM-UE)>25) who used it for 27.5 minutes (IQR: 22.3–31.5). However, a direct comparison of these two groups for device usage failed to reach significance (Z = 1.9, P = .064). Correlations of device use daily duration with the following baseline features were also non-significant: cognitive score (MoCA) (r = .39; P = .058), global neurological disability (NIHSS) (r = −.17; P = .375), age (r = −.04; P = .828), days from stroke onset (r = .03; P = .886) and enrolment duration (r = −.09; P = .628).

## Discussion

This study demonstrates that inpatient stroke survivors can achieve significant increases in exercise dose when provided with an adapted exergaming device, without requiring additional professional supervision. Participants – who accounted for 40% of stroke in-patients with UL impairment undergoing therapy – increased daily UL exercise duration 2-fold (on average), and repetition counts 8-fold, compared to conventional doses of supervised therapy.

Whilst dose was modest relative to what is considered to be therapeutic,^8^ this sample included participants with severe UL impairment (including no active movement) and this study is set in over a short duration of time, within an acute hospital setting where self-management interventions are poorly adopted due to competing acute clinical priorities,^37^ likely affecting the average supplementary UL exercise dose reported in this study. The study demonstrated that adapted exergames for self-directed UL rehabilitation were feasible, acceptable and safe; while compliance with a self-exercise guidebook (GRASP) outside of supervised sessions was poor.^38^ The relevance of these findings to everyday practice follows from the facts that we recruited a broad cohort of patients (including those with severe UL weakness and cognitive impairment) and the intervention was low cost (device plus a once-off training session). Thus, a significant proportion of stroke survivors may potentially benefit from adapted exergaming; while its adoption could occur with relatively low demands on infrastructure and resources.

The greatest reason for participant exclusion was lack of capacity to provide informed research consent (a requirement of the ethics board). Yet, some training software involved simple grip-release feedback tasks that many individuals with heightened cognitive support needs may still be able to engage in. Indeed, half of our participants had cognitive scores within the impaired range (MoCA 22/30; normal range ≥26), and there was no evidence for less device use with reduced cognitive ability. A related consideration is that 62% of participants required some level of assistance in using the device; which may partly account for more severely disabled participants showing a trend to less exergaming engagement (since they depended upon availability of relatives or incidental staff). Levels of self-directed exergaming could potentially be improved by organizational modifications such as making healthcare assistants/volunteers more available or permitting longer visitor hours.

This feasibility study did not address efficacy outcomes; the sample size was small, and the intervention/control groups were not randomly allocated, meaning that the two groups may have differed in ways other than the intervention (despite comparisons of baseline features being non-significant). However, the relevance of our findings of large increases in exercise dose between intervention and control groups lies in well-established associations between exercise dose and outcomes.^7^ This has been found across a large range of training methods,^39,40^ suggesting that these results could translate into UL improvements, if the level of exercise supplementation achieved in this study continued over a longer period. A meta-analysis of randomized controlled therapy trials^7^ found that increasing exercise dose by an average of 33 hours resulted in clinically meaningful improvements in functional outcomes. Although the median length of participation in this trial was only 8 days by comparison, it is notable that there was no trend for exergame use waning over time; with only 1 of 9 patients enrolled for at least 21 days showing a temporal drop-off.

This study does not provide evidence that UL exercises carried out with the exergaming device we used would achieve meaningful rehabilitation outcomes. The device offers training of a relatively limited number of distal UL movements, that is, a trade-off for its simplicity, accessibility and low cost. However, training of functional hand movements can ‘carry over’ improvements to more proximal UL components, and may be more instrumental than proximal/reach to grasp training.^41,42^ Extending device functionality, for example, reaching practice, could be achieved with further device development (e.g. adding a wearable armband motion sensor). We compare conventional care dose with the research intervention dose. We use the metrics of ‘time’ and ‘repetitions’ to facilitate this comparison, these metrics, however, while important, do not account for the complexity and context of rehabilitation. A further important development for future efficacy work, will involve dose quantification and articulation. Acknowledging and addressing the multidimensionality of dose is a critical component in interpreting and reproducing UL stroke recovery research.^43^

A large number of UL training technologies for stroke have been trialled over the last ten years, and so we highlight the key differences of the adapted exergaming device tested here. Robotic and virtual-reality UL training technologies have been shown in trials to be as good as, or superior to, conventional supervised therapy, when used typically at least several times a week, over 2–4 months, in sessions of 30–60 minutes, achieving 300–600 repetitions per session.^44-52^ However, in these trials, intervention sessions are generally set up and/or supervised by therapists, to encourage high-intensity training and support complex equipment set up etc.^53^ These aspects can be problematic when translating technologies to practice, because higher doses of training entails higher staffing requirements, offsetting their cost-effectiveness.^54^

To increase the likelihood for adoption, simple, portable UL training technologies have emerged, that encourage some degree of self-directed or partially self-directed exercise. However, this still leaves a burden on formal support structures. When comparing self-directed interventions it is important to consider participant characteristics, particularly age, physical and cognitive ability, which strongly influence the level of exercise self-engagement.^55^ Most studies trialling self-directed UL exergaming technologies have been highly selective, recruiting subjects typically younger, less severely affected and less cognitively impaired than the average stroke survivor, which limits their generalizability.^56-60^ For example, two studies achieving higher daily self-administered UL training doses than in our study (of 31 and 74 mins, respectively^59,61^); recruited participants ∼15 years younger than our cohort; had milder UL impairment and either needed to agree to wear a restraint mitt on their unaffected UL for the majority of the day throughout the intervention period^61^ or attend an outpatients clinic weekly,^58^ implying participants were highly motivated, with a relatively low global disability status. Many such research studies also fail to report the nature and overall number of the screening population, making it difficult to infer the impact of their findings on unselected stroke populations. By contrast since our study screened subacute stroke in-patients, we were able to quantify participant selection carefully and recruited a significantly large proportion of all UL-impaired stroke survivors, including many with severe weakness and/or cognitive impairment.

### Limitations

While we endeavoured to recruit a heterogenous, representative sample of stroke survivors, we note clear limitations in the generalizability of this feasibility work, which was conducted in a single centre, with a modest sample size. While the results we present here are promising, authors indicate there may be changes in perseverance with self-directed interventions over time.^62^ It is not clear from this work, how well stroke survivors may adhere to this intervention in the longer term. Our findings indicate that this research protocol and intervention are safe, feasible and acceptable to participants. These findings will guide further work in this area.

In summary, this is the first trial of self-directed UL exergaming intervention in an unselected inpatient stroke population, showing that exercise dose is significantly increased by provision of a simple, portable exergaming device, relative to standard care. The resource implications of the self-directed UL exergaming intervention are low, requiring one training session and a device that if loaned to multiple patients over its lifetime (for several months at a time) would cost <$100. These findings make the case for further examination of adapted, self-directed UL exergaming in a phase 2 clinical trial, over an extended stroke recovery period, spanning hospital and home. Further work is underway in the form of a pilot multicentre randomized control trial (ClinicalTrials.gov Identifier: NCT04475692).

## References

[1] Persson HC Parziali M Danielsson A Sunnerhagen KS . Outcome and upper extremity function within 72 hours after first occasion of stroke in an unselected population at a stroke unit. A part of the SALGOT study. BMC Neurol. 2012;12:162. doi:10.1186/1471-2377-12-162. 23273107 PMC3554428

[2] Rand D Eng JJ . Predicting daily use of the affected upper extremity 1 year after stroke. J Stroke Cerebrovasc Dis. 2015;24(2):274-283. doi:10.1016/j.jstrokecerebrovasdis.2014.07.039. 25533758

[3] de Sousa DG Harvey LA Dorsch S Glinsky JV . Interventions involving repetitive practice improve strength after stroke: a systematic review. J Physiother. 2018;64(4):210-221. doi:10.1016/j.jphys.2018.08.004. 30245180

[4] Thomas LH French B Coupe J , et al. Repetitive task training for improving functional ability after stroke. Stroke. 2017;48(4):e102-e103. doi:10.1161/STROKEAHA.117.016503. PMC6464929 27841442

[5] Jeffers MS Corbett D . Synergistic effects of enriched environment and task-specific reach training on poststroke recovery of motor function. Stroke. 2018;49(6):1496-1503. doi:10.1161/STROKEAHA.118.020814. 29752347

[6] Nudo RJ Wise BM SiFuentes F Milliken GW . Neural substrates for the effects of rehabilitative training on motor recovery after ischemic infarct. Science. 80-1996;272(5269):1791-1794. doi:10.1126/science.272.5269.1791. 8650578

[7] Lohse KR Lang CE Boyd LA . Is more better? Using metadata to explore dose-response relationships in stroke rehabilitation. Stroke. 2014;45(7):2053-2058. doi:10.1161/STROKEAHA.114.004695. 24867924 PMC4071164

[8] Schneider EJ Lannin NA Ada L Schmidt J . Increasing the amount of usual rehabilitation improves activity after stroke: a systematic review. J Physiother. 2016;62(4):182-187. doi:10.1016/j.jphys.2016.08.006. 27637769

[9] Mawase F Cherry-Allen K Xu J Anaya M Uehara S Celnik P . Pushing the Rehabilitation Boundaries: Hand Motor Impairment Can Be Reduced in Chronic Stroke. Neurorehabilitation Neural Repair. 2020;34(8):733-745. doi:10.1177/1545968320939563. 32845230 PMC7457456

[10] McGlinchey MP Paley L Hoffman A Douiri A Rudd AG . Physiotherapy provision to hospitalised stroke patients: analysis from the UK sentinel stroke national audit programme. European Stroke Journal. 2019;4(1):75-84. doi:10.1177/2396987318800543. 31165097 PMC6533865

[11] Sentinel Stroke National Audit Programme Clinical Results 19/20 - data.Gov.uk. https://data.gov.uk/dataset/98a9b29e-e8d1-4a4c-9987-9152c374fca6/sentinel-stroke-national-audit-programme-clinical-results-19-20. Accessed October 16, 2020.

[12] Hayward KS Brauer SG . Dose of arm activity training during acute and subacute rehabilitation post stroke: a systematic review of the literature. Clin Rehabil. 2015;29(12):1234-1243. doi:10.1177/0269215514565395. 25568073

[13] Clarke DJ Burton L-J Tyson SF , et al. Why do stroke survivors not receive recommended amounts of active therapy? Findings from the ReAcT study, a mixed-methods case-study evaluation in eight stroke units. Clin Rehabil. 2018;32(8):1119-1132. doi:10.1177/0269215518765329. 29582712 PMC6068965

[14] Lynch EA Cadilhac DA Luker JA Hillier SL . Inequities in access to inpatient rehabilitation after stroke: An international scoping review. Top Stroke Rehabil. 2017;24(8):619-626. doi:10.1080/10749357.2017.1366010. 28835194

[15] Meadmore KL Hallewell E Freeman C Hughes A-M . Factors affecting rehabilitation and use of upper limb after stroke: views from healthcare professionals and stroke survivors. Top Stroke Rehabil. 2019;26(2):94-100. doi:10.1080/10749357.2018.1544845. 30422096

[16] Da-Silva RH Moore SA Price CI . Self-directed therapy programmes for arm rehabilitation after stroke: a systematic review. Clin Rehabil. 2018;32(8):1022-1036. doi:10.1177/0269215518775170. 29756513

[17] Domínguez-Téllez P Moral-Muñoz JA Salazar A Casado-Fernández E Lucena-Antón D . Game-based virtual reality interventions to improve upper limb motor function and quality of life after stroke: systematic review and meta-analysis. Game Health J. 2020;9(1):1-10. doi:10.1089/g4h.2019.0043. 32027185

[18] Thomson K Pollock A Bugge C Brady MC . Commercial gaming devices for stroke upper limb rehabilitation: a survey of current practice. Disabil Rehabil Assist Technol. 2016;11(6):1-8. doi:10.3109/17483107.2015.1005031. 25634339

[19] Morone G Cocchi I Paolucci S Iosa M . Robot-assisted therapy for arm recovery for stroke patients: state of the art and clinical implication. Expet Rev Med Dev. 2020;17(3):223-233. doi:10.1080/17434440.2020.1733408. 32107946

[20] Laver KE Lange B George S Deutsch JE Saposnik G Crotty M . Virtual reality for stroke rehabilitation. Cochrane Database Syst Rev. 2017;2017(11):CD008349. doi:10.1002/14651858.CD008349.pub4. PMC6485957 29156493

[21] Glegg SMN Levac DE . Barriers, facilitators and interventions to support virtual reality implementation in rehabilitation: a scoping review. PM&R. 2018;10(11):1237-1251. doi:10.1016/j.pmrj.2018.07.004. 30503231 PMC6752033

[22] Everard GJ Ajana K Dehem SB Stoquart GG Edwards MG Lejeune TM . Is cognition considered in post-stroke upper limb robot-assisted therapy trials? A brief systematic review. Int J Rehabil Res. 2020;43(3):195-198. doi:10.1097/MRR.0000000000000420. 32769583

[23] Brunner I Skouen JS Hofstad H , et al. Virtual reality training for upper extremity in subacute stroke (VIRTUES). Neurology. 2017;89(24):2413-2421. doi:10.1212/WNL.0000000000004744. 29142090

[24] Laffont I Froger J Jourdan C , et al. Rehabilitation of the upper arm early after stroke: video games versus conventional rehabilitation. A randomized controlled trial. Annals of Physical and Rehabilitation Medicine. 2020;63:173-180. doi:10.1016/j.rehab.2019.10.009. 31830535

[25] Saposnik G Cohen LG Mamdani M , et al. Efficacy and safety of non-immersive virtual reality exercising in stroke rehabilitation (EVREST): a randomised, multicentre, single-blind, controlled trial. Lancet Neurol. 2016;15(10):1019-1027. doi:10.1016/S1474-4422(16)30121-1. 27365261 PMC5108052

[26] Adie K Schofield C Berrow M , et al. Does the use of Nintendo Wii SportsTM improve arm function? Trial of WiiTM in Stroke: a randomized controlled trial and economics analysis. Clin Rehabil. 2017;31(2):173-185. doi:10.1177/0269215516637893. 26975313

[27] Park M Ko M-H Oh S-W , et al. Effects of virtual reality-based planar motion exercises on upper extremity function, range of motion, and health-related quality of life: a multicenter, single-blinded, randomized, controlled pilot study. J NeuroEng Rehabil. 2019;16(1):122. doi:10.1186/s12984-019-0595-8. 31651335 PMC6813964

[28] Connelly L Jia Y Toro ML Stoykov ME Kenyon RV Kamper DG . A pneumatic glove and immersive virtual reality environment for hand rehabilitative training after stroke. IEEE Trans Neural Syst Rehabil Eng. 2010;18(5):551-559. doi:10.1109/TNSRE.2010.2047588. 20378482

[29] Rinne P Mace M Nakornchai T , et al. Democratizing neurorehabilitation: how accessible are low-cost mobile-gaming technologies for self-rehabilitation of arm disability in stroke?. PloS One. 2016;11(10):e0163413. doi:10.1371/journal.pone.0163413. 27706248 PMC5051962

[30] Benzing V Schmidt M . Exergaming for children and adolescents: strengths, weaknesses, opportunities and threats. J Clin Med. 2018;7(11):422. doi:10.3390/jcm7110422. PMC6262613 30413016

[31] Pollock A Farmer SE Brady MC , et al. Interventions for improving upper limb function after stroke. Cochrane Database Syst Rev. 2014;2014(11):CD010820. doi:10.1002/14651858.CD010820.pub2. PMC6469541 25387001

[32] Davis FD . Perceived usefulness, perceived ease of use, and user acceptance of information technology. MIS Q. 1989;13(3):319-339. doi:10.2307/249008.

[33] Bagot K Moloczij N Arthurson L , et al. Nurses’ role in implementing and sustaining acute telemedicine: a mixed‐methods, pre‐post design using an extended technology acceptance model. J Nurs Scholarsh. 2020;52(1):34-46. doi:10.1111/jnu.12509. 31508882

[34] Kowitlawakul Y . The technology acceptance model. Comput Inf Nurs. 2011;29(7):411-418. doi:10.1097/NCN.0b013e3181f9dd4a. 20975536

[35] Harris JE Eng JJ Miller WC Dawson AS . A self-administered graded repetitive arm supplementary program (GRASP) improves arm function during inpatient stroke rehabilitation. Stroke. 2009;40(6):2123-2128. doi:10.1161/STROKEAHA.108.544585. 19359633

[36] Good Clinical Practice | Research and Innovation | Imperial College London. https://www.imperial.ac.uk/research-and-innovation/research-office/research-governance-and-integrity/what-is-research-governance-and-integrity/regulatory-frameworks/good-clinical-practice/. Accessed March 19, 2021.

[37] Sadler E Wolfe CDA Jones F McKevitt C . Exploring stroke survivors' and physiotherapists' views of self-management after stroke: a qualitative study in the UK. BMJ Open. 2017;7(3):e011631. doi:10.1136/bmjopen-2016-011631. PMC5353340 28283483

[38] Connell LA McMahon NE Harris JE Watkins CL Eng JJ . A formative evaluation of the implementation of an upper limb stroke rehabilitation intervention in clinical practice: a qualitative interview study. Implement Sci. 2014;9(1):90. doi:10.1186/s13012-014-0090-3. 25112430 PMC4156624

[39] Thomson K Pollock A Bugge C Brady M . Commercial gaming devices for stroke upper limb rehabilitation: a systematic review. Int J Stroke. 2014;9(4):479-488. doi:10.1111/ijs.12263. 24661797

[40] Kwakkel G Van Peppen R Wagenaar RC , et al Effects of augmented exercise therapy time after stroke. Stroke. 2004;35:2529-2539. doi:10.1161/01.STR.0000143153.76460.7d. Lippincott Williams & Wilkins. 15472114

[41] Rodgers H Bosomworth H Krebs HI , et al. Robot assisted training for the upper limb after stroke (RATULS): a multicentre randomised controlled trial. Lancet. 2019;394(10192):51-62. doi:10.1016/S0140-6736(19)31055-4. 31128926 PMC6620612

[42] Balasubramanian S Klein J Burdet E . Robot-assisted rehabilitation of hand function. Curr Opin Neurol. 2010;23(6):661-670. doi:10.1097/WCO.0b013e32833e99a4. 20852421

[43] Hayward KS Churilov L Dalton EJ , et al. Advancing stroke recovery through improved articulation of nonpharmacological intervention dose. Stroke. 2021;52(2):761-769. doi:10.1161/STROKEAHA.120.032496. 33430635

[44] Lo AC Guarino PD Richards LG , et al. Robot-assisted therapy for long-term upper-limb impairment after stroke. N Engl J Med. 2010;362(19):1772-1783. doi:10.1056/NEJMoa0911341. 20400552 PMC5592692

[45] Flynn N Froude E Cooke D Kuys S . Repetitions, duration and intensity of upper limb practice following the implementation of robot assisted therapy with sub-acute stroke survivors: an observational study. Disabil Rehabil Assist Technol. 2020;18:1-6. doi:10.1080/17483107.2020.1807621. 32809895

[46] Perez-Marcos D Chevalley O Schmidlin T , et al. Increasing upper limb training intensity in chronic stroke using embodied virtual reality: a pilot study. J NeuroEng Rehabil. 2017;14(1):119. doi:10.1186/s12984-017-0328-9. 29149855 PMC5693522

[47] Baniña MC Molad R Solomon JM , et al. Exercise intensity of the upper limb can be enhanced using a virtual rehabilitation system. Disabil Rehabil Assist Technol. 2020:18:1-7. doi:10.1080/17483107.2020.1765421. 32421460

[48] Demers M Chan Chun Kong D Levin MF . Feasibility of incorporating functionally relevant virtual rehabilitation in sub-acute stroke care: perception of patients and clinicians. Disabil Rehabil Assist Technol. 2019;14(4):361-367. doi:10.1080/17483107.2018.1449019. 29526122

[49] Johnson L Bird M-L Muthalib M Teo W-P . An innovative STRoke Interactive Virtual thErapy (STRIVE) online platform for community-dwelling stroke survivors: a randomised controlled trial. Arch Phys Med Rehabil. 2020;101:1131-1137. doi:10.1016/j.apmr.2020.03.011. 32283048

[50] Zhang T Wang Z-r. Wang P Xing L Mei L-p. Zhao J . Leap Motion-based virtual reality training for improving motor functional recovery of upper limbs and neural reorganization in subacute stroke patients. Neural Regeneration Research. 2017;12(11):1823-1831. doi:10.4103/1673-5374.219043. 29239328 PMC5745836

[51] Kiper P Szczudlik A Agostini M , et al. Virtual reality for upper limb rehabilitation in subacute and chronic stroke: a randomized controlled trial. Arch Phys Med Rehabil. 2018;99(5):834-842. e4. doi:10.1016/j.apmr.2018.01.023. 29453980

[52] Kim W-S Cho S Park SH Lee J-Y Kwon S Paik N-J . A low cost kinect-based virtual rehabilitation system for inpatient rehabilitation of the upper limb in patients with subacute stroke. Medicine. 2018;97(25):e11173. doi:10.1097/MD.0000000000011173. 29924029 PMC6034563

[53] Rémy-Néris O Le Jeannic A Dion A , et al. Additional, mechanized upper limb self-rehabilitation in patients with subacute stroke: the REM-AVC randomized trial. Stroke. 2021;52(6):1938-1947. doi:10.1161/STROKEAHA.120.032545. 33910364

[54] Winser S Lee SH Law HS Leung HY Bello UM Kannan P . Economic evaluations of physiotherapy interventions for neurological disorders: a systematic review. Disabil Rehabil. 2020;42(7):892-901. doi:10.1080/09638288.2018.1510993. 30616401

[55] Chi N-F Huang Y-C Chiu H-Y Chang H-J Huang H-C . Systematic review and meta-analysis of home-based rehabilitation on improving physical function among home-dwelling patients with a stroke. Arch Phys Med Rehabil. 2020;101(2):359-373. doi:10.1016/j.apmr.2019.10.181. 31689417

[56] Wittmann F Held JP Lambercy O , et al. Self-directed arm therapy at home after stroke with a sensor-based virtual reality training system. J NeuroEng Rehabil. 2016;13(1):75. doi:10.1186/s12984-016-0182-1. 27515583 PMC4982313

[57] Nijenhuis SM Prange GB Amirabdollahian F Sale P Infarinato F Nasr N , et al. Feasibility study into self-administered training at home using an arm and hand device with motivational gaming environment in chronic stroke. J NeuroEng Rehabil 2015;12(1). doi:10.1186/s12984-015-0080-y. PMC4599772 26452749

[58] Slijper A Svensson KE Backlund P Engström H Sunnerhagen K . Computer game-based upper extremity training in the home environment in stroke persons: A single subject design. J NeuroEng Rehabil. 2014;11(1):35. doi:10.1186/1743-0003-11-35. 24625289 PMC3995595

[59] Dodakian L McKenzie AL Le V , et al. A home-based telerehabilitation program for patients with stroke. Neurorehabilitation Neural Repair. 2017;31(10-11):923-933. doi:10.1177/1545968317733818. 29072556 PMC5734923

[60] Zondervan DK Friedman N Chang E Zhao X Augsburger R Reinkensmeyer DJ , et al. Home-based hand rehabilitation after chronic stroke: randomized, controlled single-blind trial comparing the MusicGlove with a conventional exercise program. J Rehabil Res Dev. 2016;53(4):457-472. doi:10.1682/JRRD.2015.04.0057. 27532880

[61] Kelly KM Borstad AL Kline D Gauthier LV . Improved quality of life following constraint-induced movement therapy is associated with gains in arm use, but not motor improvement. Top Stroke Rehabil. 2018;25(7):467-474. doi:10.1080/10749357.2018.1481605. 30246613 PMC6359892

[62] Neibling BA Jackson SM Hayward KS Barker RN . Perseverance with technology-facilitated home-based upper limb practice after stroke: a systematic mixed studies review. J NeuroEng Rehabil. 2021;18(1):43. doi:10.1186/s12984-021-00819-1. 33627126 PMC7905577

